# Watching hair turn grey

**DOI:** 10.7554/eLife.70584

**Published:** 2021-06-30

**Authors:** Michael P Philpott

**Affiliations:** Centre for Cell Biology and Cutaneous Research, Blizard Institute, Barts and The London School of Medicine and Dentistry, Queen Mary UniversityLondonUnited Kingdom

**Keywords:** hair greying, reversal, psychological stress, mitochondria, Human

## Abstract

Analysing changes in hair pigmentation may lead to a better understanding of the impacts of ‘life events’ on human biology and aging.

**Related research article** Rosenberg AM, Rausser S, Ren J, Mosharov EV, Sturm G, Ogden RT, Patel P, Kumar Soni R, Lacefield C, Tobin DJ, Paus R, Picard M. 2021. Quantitative mapping of human hair greying and reversal in relation to life stress. *eLife* **10**:e67437. doi: 10.7554/eLife.67437

When the French queen, Marie Antoinette, was led to the guillotine at the age of 37, it was said that her hair had turned white the night before. While public executions are, thankfully, extremely rare events, most of us will experience our hair turning grey or white as we get older (Tobin, 2011). However, the physiological and psychological reasons for this phenomenon are not fully understood.

All mammals have hair and its many roles include thermal regulation, physical protection and sensing various signals and stimuli (Schneider et al., 2009). Humans also seem to spend a disproportionate amount of time worrying about the appearance of their hair (Ebling, 1976).

Hair fibres grow from mini-organs called hair follicles that are found in the skin (Schneider et al., 2009). At any given time, a hair follicle will be in one of the three stages of the hair growth cycle: a period of active growth (anagen), a period of regression (catagen), or a period of rest (telogen; Oh et al., 2016). In humans this cycle can last for as little as three months (in the eyebrow) or for as long as several years (in the scalp). Hair colour comes from pigments called melanin that are produced inside the follicle during anagen by cells called melanocytes (Slominski et al., 2005).

Now, in eLife, Martin Picard (Columbia University and the New York State Psychiatric Institute) and colleagues – including Ayelet Rosenberg as first author – report that they have developed a computational model that can relate changes in hair pigmentation to ‘life events’ (Rosenberg et al., 2021). This work will allow researchers to map the stressful life events that turn hair grey, contributing to a greater understanding of the effects of stress and other experiences on human biology (Figure 1).

**Figure 1.**
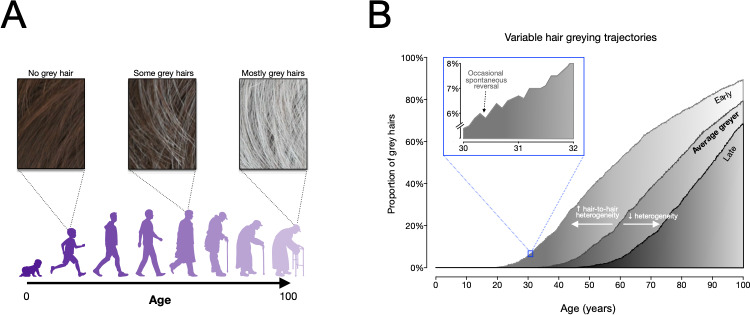
Modelling human hair greying. (**A**) An overview of how hair greys as people age, from having no grey hairs when people are children and young (left), to having a full head of grey as people get older (right). The model developed by Rosenberg et al. is consistent with this pattern. (**B**) Frequency distributions of grey hairs for individuals with early (light grey), average (medium grey) and late (dark grey) onset hair greying. The model predicts that increased heterogeneity of hair greying between individual hair follicles can explain early greying, whilst decreased heterogeneity can explain late onset greying. The inset (framed in blue) shows that the model accurately predicts the temporary reversals of greying observed by Rosenberg et al. Figure adapted from Figure 5 in Rosenberg et al., 2021.

Rosenberg et al. first developed methods to digitally map hair pigment patterns across the length of a hair fibre. Since the daily rate of hair growth was already known, the researchers could relate changes in pigmentation to a specific time. This opens up the possibility of using changes in pigmentation to learn about life events experienced by the owner of the hair, analogous to the way that tree rings can be used to provide information about atmospheric conditions in the past. Building on previous work (Adav et al., 2018), Rosenberg et al. also carried out a detailed proteomic analysis of dark and white hair, and identified over 300 proteins that were more or less abundant in white hair than in dark hair. Many of these proteins are involved in energy production in the cell: white hair fibres contained more proteins associated with mitochondria, more proteins involved in increasing energy metabolism, and more proteins with a role in synthesising lipids and amino acids.

Many of the proteins found at higher levels in white hair are known to be rapidly remodelled by environmental and neuroendocrine factors, which suggests that the process of hair turning grey may be more ‘plastic’ than previously thought, and may even be reversible. To investigate this, Rosenberg et al. studied 14 individuals with hair that showed evidence of re-pigmentation and, in some cases, re-pigmentation followed by a second loss of colour. These changes occurred within one anagen and were often synchronised between a number of hair fibres, suggesting that going grey and re-pigmentation are regulated by cellular mechanisms. Moreover, the rate of greying was fast, with up to 14% of hair colour being lost each day; and re-pigmentation was even faster, occurring at twice this rate. One possible mechanism to explain re-pigmentation involves the activation of either immature melanocytes or melanoblasts (melanocyte precursor cells) from outside of the hair follicle. These cells would then migrate into the hair follicle and produce melanin, re-colouring the hair.

Rosenberg et al. then investigated whether going grey and re-pigmentation were linked to psychosocial stress, and whether specific proteomic signatures were associated with greying. First, they used the fact that changes in hair pigmentation can be correlated to time to show that sudden changes in hair pigmentation are associated with changes in stress levels as reported by the participants in the study. Next, Rosenberg et al. showed that re-pigmentation correlated with perceived decreased stress. Having established that hair pigmentation stress mapping can be used to correlate greying with stress, the researchers re-analysed their protein data sets and found that greying is linked to mitochondrial metabolism.

In a final step, Rosenberg et al. developed a mathematical model that could simulate the greying of an entire head of hair over time. The model includes an aging factor, a biological threshold and a stress factor; and uses basic tenets regarding the age of onset of hair greying, its progressive nature, and the fact that as a person gets older, more of their follicles go grey. It also takes into account the fact that age related greying may be reversible and involve life events.

Much more work will be required to validate this model, but the experiments of Rosenberg, Picard, and colleagues – who are based at Columbia and other institutions in the US, Germany and Ireland – provide an excellent basis from which hair pigmentation can be mapped and modelled. This can begin to explain how ‘life stress events’ impact human bodies. However, the latest findings might also have an impact that extends beyond the development of tools to understand the effect of stress on human biology: in particular, the latest work suggests that human aging may not be a linear, fixed biological process but may, at least in part, be halted or even temporarily reversed.
